# Activation of AMP-activated protein kinase rapidly suppresses multiple pro-inflammatory pathways in adipocytes including IL-1 receptor-associated kinase-4 phosphorylation

**DOI:** 10.1016/j.mce.2016.11.010

**Published:** 2017-01-15

**Authors:** Sarah J. Mancini, Anna D. White, Silvia Bijland, Claire Rutherford, Delyth Graham, Erik A. Richter, Benoit Viollet, Rhian M. Touyz, Timothy M. Palmer, Ian P. Salt

**Affiliations:** a Institute of Cardiovascular and Medical Sciences, College of Medical, Veterinary and Life Sciences, University of Glasgow, Glasgow, G12 8QQ, United Kingdom; b Section of Molecular Physiology, Department of Nutrition, Exercise and Sports, Faculty of Science, University of Copenhagen, Denmark; c INSERM, U1016, Institut Cochin, Paris, France; d CNRS, UMR8104, Paris, France; e Université Paris Descartes, Sorbonne Paris Cité, France; f School of Pharmacy, University of Bradford, Bradford, West Yorkshire, BD7 1DP, United Kingdom

**Keywords:** Adipocyte, AMP-activated protein kinase, Inflammation, Signalling

## Abstract

Inflammation of adipose tissue in obesity is associated with increased IL-1β, IL-6 and TNF-α secretion and proposed to contribute to insulin resistance. AMP-activated protein kinase (AMPK) regulates nutrient metabolism and is reported to have anti-inflammatory actions in adipose tissue, yet the mechanisms underlying this remain poorly characterised. The effect of AMPK activation on cytokine-stimulated proinflammatory signalling was therefore assessed in cultured adipocytes. AMPK activation inhibited IL-1β-stimulated CXCL10 secretion, associated with reduced interleukin-1 receptor associated kinase-4 (IRAK4) phosphorylation and downregulated MKK4/JNK and IKK/IκB/NFκB signalling. AMPK activation inhibited TNF-α-stimulated IKK/IκB/NFκB signalling but had no effect on JNK phosphorylation. The JAK/STAT3 pathway was also suppressed by AMPK after IL-6 stimulation and during adipogenesis. Adipose tissue from *AMPKα1*^−/−^ mice exhibited increased JNK and STAT3 phosphorylation, supporting suppression of these distinct proinflammatory pathways by AMPK *in vivo*. The inhibition of multiple pro-inflammatory signalling pathways by AMPK may underlie the reported beneficial effects of AMPK activation in adipose tissue.

## Highlights

• AMPK rapidly inhibits proinflammatory NFκB and JNK signalling in adipocytes.
• Mechanisms underlying AMPK-induced effects involve inhibition of IRAK4, IKK and MKK4 phosphorylation.
• AMPK concurrently inhibits JAK-STAT signalling in adipocytes.
• Adipose tissue from mice lacking AMPKα1 exhibit increased proinflammatory signalling.

## Graphical abstract

**Figure:**
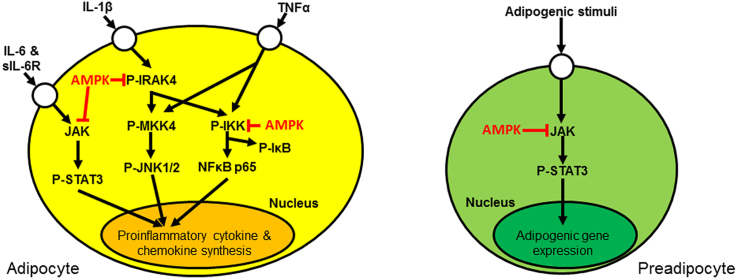

## Abbreviations

ACC: acetyl-CoA carboxylase
Ad.AMPK-CA: adenoviruses expressing constitutively active mutant AMPKα1
Ad.α1DN: adenoviruses expressing dominant negative mutant AMPKα1
Ad.GFP: adenoviruses expressing green fluorescent protein
AMPK: AMP-activated protein kinase
CEBP: CAAT-enhancer-binding protein
CXCL: chemokine (CXC motif) ligand
GAPDH: glyceraldehyde-3-phosphate dehydrogenase
IκB: inhibitor of NFκB
IKK: IκB kinase
IRAK4: interleukin-1 receptor associated kinase-4
JAK: Janus kinase
JNK: *c-jun* N-terminal kinase
MAPK: mitogen-activated protein kinase
MCP-1: monocyte chemoattractant protein-1
MKK: MAPK kinase
MKP-1: MAPK phosphatase-1
NFκB: nuclear factor κB
sIL-6Rα: soluble IL-6 receptor-α
PPARγ: peroxisome proliferator-activated receptor-γ
STAT: signal transducer and activator of transcription
WAT: white adipose tissue

## 1 Introduction

Obesity is associated with chronic low-grade inflammation of white adipose tissue (WAT), in which there is upregulated secretion of the pro-inflammatory cytokines TNF-α, IL-1β and IL-6. Plasma concentrations of these pro-inflammatory cytokines have been reported to be elevated in obese individuals (Lee et al., 2009). TNF-α and IL-1β trigger pro-inflammatory effects *via* simultaneous activation of the nuclear factor kappa B (NFκB) and multiple mitogen-activated protein kinase (MAPK) intracellular signalling pathways. IL-1β binding leads to recruitment of adapter proteins that further recruit IL-1 receptor associated kinase (IRAK) family members, directly interacting with IRAK4 which autophosphorylates and activates the downstream kinases IRAK1 and IRAK2. IRAK activation further stimulates formation of a signalosome including TNF receptor-associated factor-6 (TRAF6), TGFβ-activated kinase-1 (TAK1) and IκB kinase (IKK) (Salt and Palmer, 2012). TNF-α engages multiple complex ubiquitin-dependent processes that also stimulate TAK1 and IKK (Salt and Palmer, 2012). IKK stimulates the phosphorylation of inhibitor of NFκB (IκBα), targeting IκB for proteasomal degradation and thereby releasing active NFκB dimers, with p65-p50 heterodimers being the principal form activated by the canonical NFκB pathway in response to TNF-α and IL-1β (Cohen, 2014, Brenner et al., 2015, Salt and Palmer, 2012). The p65-p50 heterodimers subsequently translocate into the nucleus to bind κB-responsive elements in the promoters of target genes, including other pro-inflammatory cytokines and chemokines. TNF-α and IL-1β stimulate activation of pro-inflammatory MAPKs, such as the *c-jun* N-terminal kinase (JNK) pathway, in parallel with NFκB activation (Cohen, 2014, Salt and Palmer, 2012).

In contrast, IL-6 elicits its effects *via* binding a membrane-bound IL-6 receptor complexed with the co-receptor gp130 (classic signalling), or by binding a soluble IL-6 receptor (sIL-6Rα) that binds to gp130 (*trans*-signalling), stimulating Janus kinase (JAK)-mediated activation of the signal transducer and activator of transcription-3 (STAT3) transcription factor predominantly *via* phosphorylation of Tyr705 (Salt and Palmer, 2012, Qu et al., 2014). The cytokine-stimulated NFκB, JNK and JAK/STAT pathways culminate in the secretion of multiple pro-inflammatory cytokines and chemokines, including monocyte chemoattractant protein-1 (MCP-1), which stimulates the activation and infiltration of macrophages (Cohen, 2014, Brenner et al., 2015; Salt and Palmer, 2012, Qu et al., 2014). In adipose tissue, macrophages undergo a shift in polarisation from an anti-inflammatory ‘alternatively activated’ state, to a ‘classically activated’ pro-inflammatory state, which has been reported to dominate in WAT in obesity (Lumeng et al., 2007).

Increased ATP consumption and/or decreased ATP synthesis, leads to an increase in ADP:ATP and AMP:ATP ratios that activate the heterotrimeric Ser/Thr protein kinase AMP-activated protein kinase (AMPK) (Salt and Palmer, 2012, Bijland et al., 2013). Activated AMPK phosphorylates target proteins that act to normalise ATP concentrations by stimulating ATP-generating pathways such as fatty acid oxidation, mitochondrial biogenesis and muscle glucose transport, whilst inhibiting ATP-consuming anabolic pathways including protein translation, fatty acid and cholesterol synthesis (Salt and Palmer, 2012, Bijland et al., 2013). In adipose tissue, AMPK suppresses adipogenesis, fatty acid and triglyceride synthesis (Bijland et al., 2013).

More recently, AMPK has been reported to have anti-inflammatory actions independent of its metabolic effects, diminishing immune responses in a range of *in vivo* models of inflammation (Salt and Palmer, 2012). Although AMPK-dependent inhibition of pro-inflammatory signalling has been reported in preadipocytes, macrophages and vascular cells (Jeong et al., 2009, Sag et al., 2008, Ewart et al., 2008, Chen et al., 2016), all of which are present in significant quantities in intact adipose tissue, AMPK-dependent suppression of pro-inflammatory signalling in adipocytes remains poorly characterised. Various AMPK activators have been reported to reduce pro-inflammatory cytokine expression and NFκB activation in adipose tissue from rodents (Jeong et al., 2009, Sun et al., 2014) and inhibit expression and secretion of pro-inflammatory cytokines in human subcutaneous WAT cultured *ex-vivo* (Lihn et al., 2004, Lihn et al., 2008). Importantly, however, these studies did not examine the effect of AMPK activation in adipocytes themselves or demonstrate the AMPK-dependence of the AMPK activators used. This is critical due to the non-specific nature of and AMPK-independent effects reported for several widely-used AMPK activators, including AICAR, phenformin and metformin (García-García et al., 2010, Guigas et al., 2006). Three studies have reported inhibition of IKKβ phosphorylation in cultured adipocytes or rodent adipose tissue by AMPK activators in a manner sensitive to compound C, which has been used widely to imply AMPK-dependence (Wang et al., 2013, Zhao et al., 2014, Chen et al., 2016). *Compound C is a poorly*-*selective inhibitor of AMPK*, *with* many reported AMPK-independent effects and inhibiting several protein kinases other than AMPK with similar or greater potency (Salt and Palmer, 2012, Bain et al., 2007)*. Indeed, compound C has been demonstrated to influence pro-inflammatory signalling independent of AMPK* (Kim et al., 2011).

As AMPK is activated by several existing drugs used to treat type 2 diabetes, including metformin and thiazolidinediones (Bijland et al., 2013), there is a need to understand the actions of AMPK activation in metabolic tissues. Therefore, given the proposed anti-inflammatory actions of AMPK and dearth of information concerning the role of AMPK in adipocyte pro-inflammatory signalling, we investigated the effect of AMPK activation on pro-inflammatory signalling in response to IL-1β, TNF-α and IL-6, the principal cytokines associated with adipose tissue inflammation, in cultured adipocytes using the selective AMPK activator A769662, which directly binds and activates AMPK heterotrimers containing the β1 non-catalytic subunit (Scott et al., 2008). We also examined basal pro-inflammatory signalling pathway status in adipose tissue lacking the AMPKα1 catalytic subunit.

## 2 Materials and methods

### 2.1 Materials

3T3-L1 preadipocytes which overexpress the coxsackie virus and adenovirus receptor (3T3-L1Δ1CAR) (Ross et al., 2003) were a kind gift from Prof. David Orlicky, University of Colorado (CO, USA). Adenoviruses expressing c-myc-tagged dominant negative mutant AMPKα1 (Ad.α1-DN), constitutively active AMPKα1 (Ad.AMPK-CA), GFP (Ad.GFP) and control adenoviruses (Ad.null) have been described previously (Woods et al., 2000), and were a generous gift from Dr F. Foufelle, Centre Biomédical des Cordeliers, Paris. A769662 was obtained from Abcam (Cambridge, UK). [γ-^32^P]-ATP was obtained from Perkin Elmer (Buckinghamshire, UK). SAMS peptide was obtained from GL Biochem Ltd (Shanghai, China). Mouse IL-6 and mouse soluble IL-6 receptor-α (sIL-6Rα) were obtained from R&D Systems (Abingdon, Oxfordshire, UK). Mouse TNF-α and IL-1β were obtained from Sigma (Dorset, UK). Rabbit anti-phospho-acetyl CoA carboxylase (ACC) S79, anti-phospho-AMPK T172, anti-AMPKα, anti-JNK, anti-phospho-c-Jun S63, anti-c-Jun, anti-phospho IκB S32, anti-phospho-IRAK4 T345/S346, anti-IRAK4, anti-phospho-MKK4 S257, anti-MKK4, anti-STAT3, anti-phospho IKKα/β S176/S177, anti-IKKβ, anti-NFκB p65 antibodies and mouse anti-phospho-JNK T183/Y185, anti-phospho-STAT3 Y705 and anti-IκB antibodies were from New England Biolabs (Hitchin, Hertfordshire, UK). Mouse anti-myc (9E10) antibodies were from Santa Cruz Biotechnology (California, US). Mouse anti-GAPDH was obtained from Ambion (Cambridge, UK). Sheep anti-ACC, anti-AMPKα1 and anti-AMPKα2 antibodies were a kind gift from Prof. D. G. Hardie, University of Dundee (Dundee, UK). Donkey Infrared dye-labelled secondary antibodies were from LI-Cor Biosciences (Cambridge, UK). Goat anti-rabbit Alexa Fluor 488 secondary antibody was from Invitrogen (Paisley, UK). Mouse cytokine 20-plex panel for Luminex platform was obtained from Invitrogen (Paisley, UK). All other reagents were from sources described previously (Ewart et al., 2008, Boyle et al., 2011).

### 2.2 Cell culture

3T3-L1 and 3T3-L1Δ1CAR fibroblasts were cultured and differentiated into adipocytes as described previously (Boyle et al., 2011). Human SW872 cells were cultured and differentiated into adipocytes as described previously (Rios et al., 2015). Mouse embryonic fibroblasts (MEFs) lacking AMPKα1 and AMPKα2 (Laderoute et al., 2006) and wild-type control MEFs were cultured in DMEM and 10% (v/v) FCS. 3T3-L1 preadipocytes were used between passages 8 and 12 and 8–12 days post differentiation. 3T3-L1Δ1 CAR preadipocytes were used between passages 15 and 30 and 8–12 days post differentiation. SW872 adipocytes were stimulated on day 9 post-differentiation having been serum-deprived overnight in DMEM and 1% (v/v) NCS.

### 2.3 Preparation of adenoviruses and infection of 3T3-L1Δ1CAR adipocytes

Ad.α1-DN, Ad.AMPK-CA, Ad.GFP and Ad.null were propagated, purified and titred as described previously (Ewart et al., 2008). 3T3-L1Δ1CAR adipocytes were infected on day 6 post-differentiation with adenoviruses in serum-free DMEM. After incubation for 4 h, DMEM supplemented with 20% (v/v) FCS was added to achieve 10% (v/v) final concentration and the cells incubated for a further 48 h prior to preparation of cell lysates.

### 2.4 Confocal immunofluorescence microscopy

Coverslips of 3T3-L1 or 3T3-L1Δ1CAR adipocytes were washed with PBS and fixed in 3% (w/v) paraformaldehyde at room temperature for 20 min. Coverslips were then washed twice in PBS and twice in 20 mmol/l glycine in PBS. Cells were incubated in permeabilisation media (PBS containing 2% (w/v) BSA, 0.1% (w/v) saponin, 20 mmol/l glycine) for 20 min. Coverslips were incubated in primary antibodies in permeabilisation media for 45 min, washed in permeabilisation media four times and further incubated in fluorophore-conjugated secondary antibodies in permeabilisation media for 30 min. Cells were washed four times in permeabilisation media, once in PBS and mounted on microscope slides prior to visualisation on a Zeiss LSM 5 Pa Exciter laser scanning microscope.

### 2.5 Analysis of cytokine/chemokine production

3T3-L1Δ1CAR adipocytes infected with adenoviruses were incubated in serum-free DMEM and stimulated with 10 ng/ml IL-1β or a sIL-6Rα/IL-6 (25 ng/ml and 5 ng/ml, respectively) *trans*-signalling complex for 6 h with or without 30 min preincubation with 300 μmol/l A769662. After this incubation period, cells were washed three times in serum-free DMEM and incubated with 0.4 ml/well DMEM for 1 h at 37 °C, 10% (v/v) CO_2_. After 1 h, the conditioned serum-free DMEM was collected and assayed for cytokine/chemokine content using a Luminex mouse chemokine 20-plex bead immunoassay kit and a Luminex 100™ detection system (Life Technologies, Paisley, UK), testing for the presence of the cytokines GM-CSF, IFN-γ, IL-1α, IL-1β, IL-2, IL-4, IL-5, IL-6, IL-10, IL-12, IL-13, IL-17 and TNF-α, the chemokines, CXCL1, CXCL9, CXCL10 and MCP-1, basic fibroblast growth factor (bFGF) and vascular endothelial growth factor (VEGF).

### 2.6 RNA extraction from SW872 adipocytes and gene expression analysis

RNA was extracted from SW872 adipocytes using an RNeasy kit (Qiagen). Between 400 and 1000 ng of RNA was reverse-transcribed using the High Capacity cDNA Reverse Transcription kit (Applied Biosystems). qPCR was performed with an Applied Biosystems ABI-PRISM 7900HT Sequence Detection System. Gene expression was normalised to TATA binding protein (TBP) using Assays on Demand and QPCR master mix (Applied Biosystems). The following TaqMan^®^ Gene Expression Assays (Applied Biosystems) were used: TBP (Hs00427620_m1) and CXCL10 (Hs01124251_g1).

### 2.7 Preparation of cell lysates, SDS PAGE and immunoblotting

Cell lysates were prepared, proteins resolved by SDS-PAGE and subjected to quantitative immunoblotting with the antibodies indicated as described previously (Boyle et al., 2011). Immunolabelled proteins were visualized using infrared dye-labelled secondary antibodies and an Odyssey Sa infrared imaging system (LiCor Biosciences UK Ltd, Cambridge, UK). Band density was quantitated with Image J software.

### 2.8 Immunoprecipitation and assay of AMPK activity

AMPK was immunoprecipitated from 3T3-L1 adipocyte lysates using a mixture of sheep anti-AMPKα1 and anti-AMPKα2 antibodies and assayed using the SAMS substrate peptide as previously described (Boyle et al., 2011).

### 2.9 Oil red O staining of 3T3-L1 adipocytes

3T3-L1 adipocytes cultured on glass coverslips were incubated in the presence or absence of A769662 (300 μmol/l). At various intervals from the point of differentiation, cells were fixed using 10% (v/v) formalin for 1 h, then washed once in 60% (v/v) isopropanol and left to dry completely. Coverslips were incubated in 5.1 mmol/l Oil Red O in 60% (v/v) isopropanol for 10 min before washing four times with dH_2_0 and left to dry. Dry coverslips were then dipped in Mayers Hematoxylin (194 mmol/l KAl(SO_4_)_2_, 16.5 mmol/l hematoxylin, 2 mmol/l NaIO_3_, 2% (v/v) acetic acid), followed by 3% (v/v) NH_4_OH for 10 s. Coverslips were mounted onto slides and images captured using an Axiovision light microscope.

### 2.10 Animals

Female AMPKα1^−/−^ and wild-type sv129 mice (Jørgensen et al., 2004) were housed in a 12-h light dark cycle with access to food (normal chow diet) and water ad libitum. All experimental procedures were performed in accordance with UK Home Office Guidance on the operation of the Animals (Scientific Procedures) Act (1986), the “Guide for the Care and Use of Laboratory Animals” published by the US National Institutes of Health (eighth edition) and institutional ethical approval (PPL 70/8572, PPL60/4224).

### 2.11 Glucose tolerance test

Intraperitoneal glucose tolerance test was performed on 18–20 week old mice. Mice were fasted for 16 h prior to 1 g/kg intraperitoneal injection of D-glucose in 0.9% saline (Sigma). Blood glucose levels were measured from tail samples with a glucometer (Freestyle Optium Xceed; Abbott, Berkshire,UK) at 0, 15, 30, 60 90 and 120 min.

### 2.12 Preparation of adipose tissue lysates

Mice (aged 18–20 weeks) were killed by cervical dislocation. Subcutaneous and gonadal adipose tissue was excised and snap-frozen prior to preparation of lysates as described previously (Boyle et al., 2011).

### 2.13 Statistics

Results are expressed as mean ± SEM. Statistically significant differences were determined using a two-tail *t*-test, or one or two-way ANOVA where appropriate, with *p* < 0.05 as significant.

## 3 Results

**Fig. 1:**
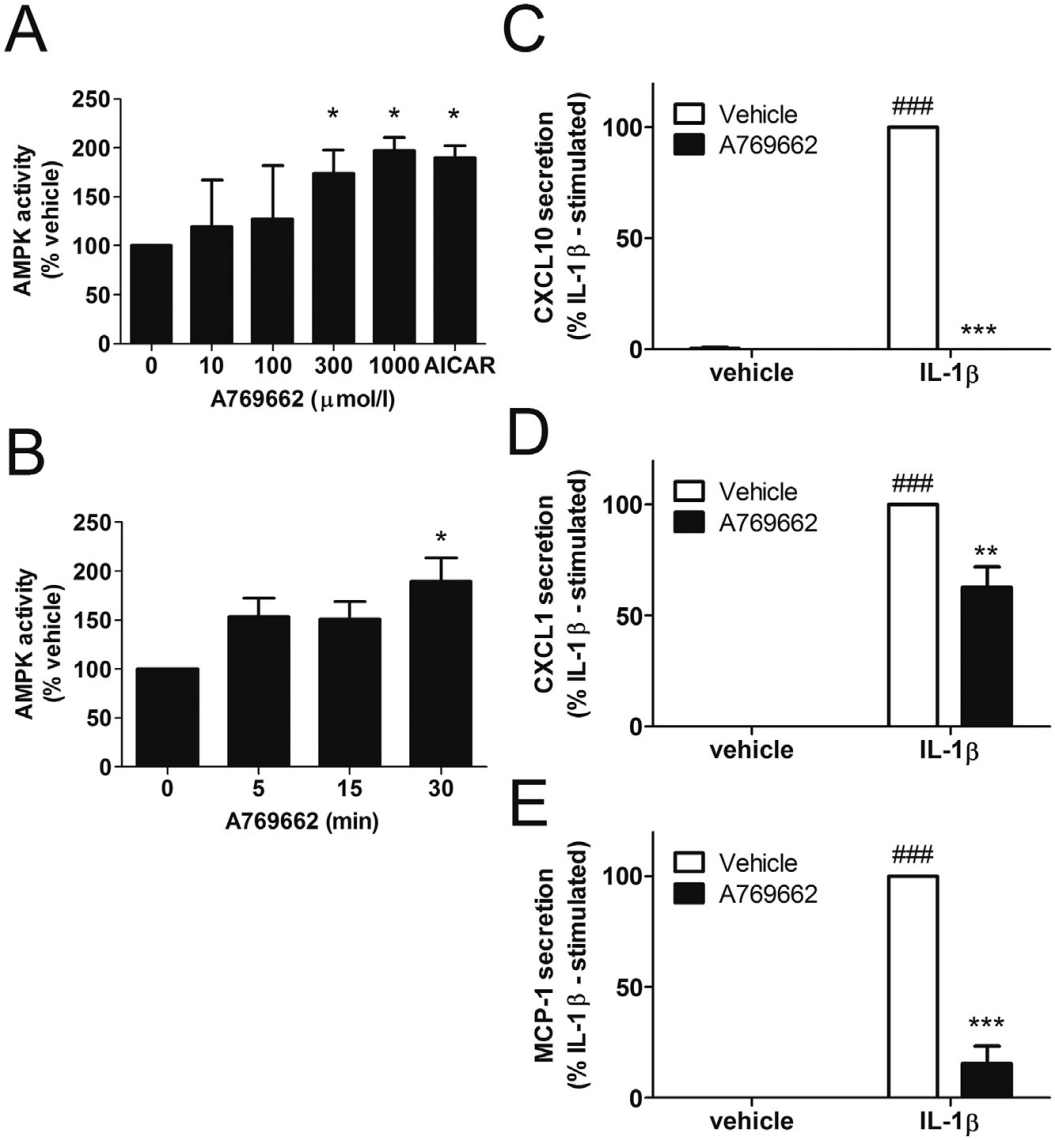
AMPK activation suppresses IL-1β-stimulated chemokine secretion. 3T3-L1 adipocytes were (A) stimulated with the indicated concentrations of A769662 for 30 min or 1 mmol/l AICAR for 60 min and lysates prepared or (B) stimulated with 300 μmol/l A769662 for the indicated durations. AMPK was immunoprecipitated from lysates and assayed for AMPK activity. Data shown represents AMPK activity (% vehicle) from 3 independent experiments. ^∗^p < 0.05 vs vehicle (two-tail *t*-test). (C–E) 3T3-L1Δ1CAR adipocytes infected with Ad.Null were preincubated for 30 min in the presence or absence of A769662 (300 μmol/l) prior to stimulation in the presence or absence of IL-1β (10 ng/ml) for 6 h. Conditioned medium was collected and chemokine secretion assayed using a multiplex bead immunoassay. Data shown represents IL-1β-stimulated (C) CXCL10 (D) CXCL1 or (E) MCP-1 secretion from three independent experiments. ^###^p < 0.001 vs absence of IL-1β. **p < 0.01, ***p < 0.001 vs absence of A769662 (one way ANOVA).

**Fig. 2:**
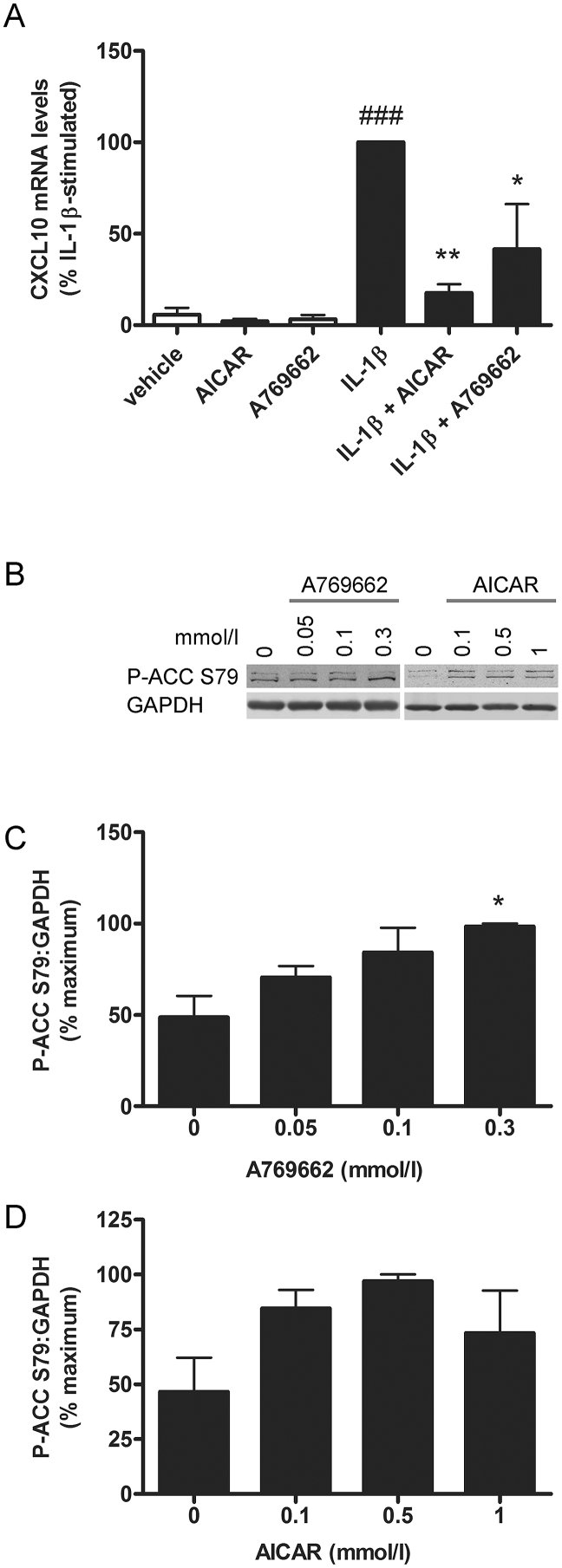
AMPK activators suppress IL-1β-stimulated CXCL10 mRNA expression in human SW872 adipocytes. (A) Human SW872 adipocytes were stimulated with IL-1β (10 ng/ml) for 6 h following preincubation for 30 min in the presence or absence of A769662 (0.1 mmol/l) or AICAR (1 mmol/l) and chemokine mRNA levels analysed by qPCR. Data shown represents the % IL-1β-stimulated CXCL10 mRNA expression normalised to TATA-binding protein from three independent experiments. ###p < 0.001 relative to absence of IL-1β. **p < 0.01, *p < 0.05 relative to IL-1β alone (one-way ANOVA). (B–D) SW872 adipocytes were stimulated with the indicated concentrations of A769662 or AICAR for 30 min and lysates prepared. Lysate proteins were resolved by SDS-PAGE and subjected to immunoblotting with the antibodies indicated. (B) Representative immunoblots repeated on two further occasions with similar results. (C, D) Densitometric analysis of ACC phosphorylation relative to GAPDH levels from three independent experiments. *p < 0.05 relative to absence of A769662 (one-way ANOVA).

Stimulation of 3T3-L1 adipocytes with 300–1000 μmol/l A769662 or 1 mmol/l AICAR significantly stimulated AMPK activity (Fig. 1A), such that 300 μmol/l was used for subsequent experiments. The stimulatory effect of A769662 was observed after 30 min incubation (Fig. 1B) To determine the effect of A769662 on chemokine secretion, 3T3-L1Δ1CAR adipocytes were stimulated with IL-1β at a concentration previously reported to stimulate IL-13 expression in 3T3-L1 adipocytes (Kwon et al., 2014) and chemokine/cytokine secretion assessed using a murine chemokine multiplex bead immunoassay and Luminex^®^ 100™ detection system. Of the 20 cytokines and chemokines assayed, IL-1β stimulation markedly increased secretion of the chemokines CXCL1, CXCL10 and MCP-1. There was no detectable secretion of GM-CSF, IFN-γ, IL-1α, IL-1β, IL-2, IL-4, IL-5, IL-6, IL-10, IL-12, IL-13, IL-17, TNF-α, CXCL9, bFGF or VEGF in conditioned media from 3T3-L1Δ1CAR adipocytes under basal or IL-1β-stimulated conditions. Intriguingly, A769662 abolished IL-1β-stimulated secretion of CXCL10, markedly reduced MCP-1 secretion and modestly reduced IL-1β-stimulated CXCL1 secretion (Fig. 1C–E). Furthermore, pre-incubation of human SW872 adipocytes with A769662 or the chemically-unrelated AMPK activator AICAR attenuated IL-1β-stimulated CXCL10 mRNA expression, at concentrations that activated AMPK activity, as assessed by phosphorylation of the AMPK substrate, ACC (Fig. 2).

**Fig. 3:**
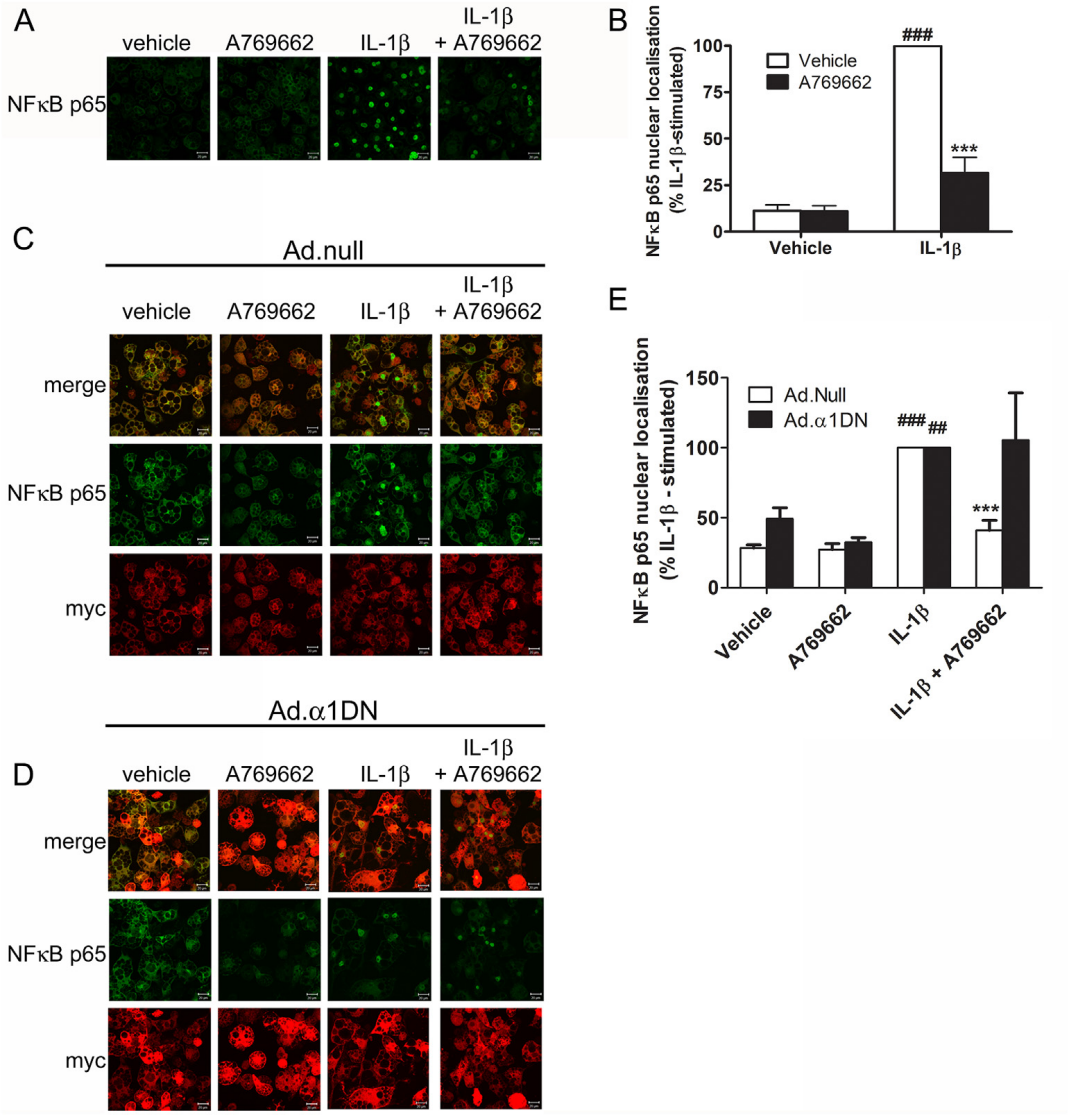
AMPK activation inhibits IL-1β-stimulated NFκB p65 nuclear translocation. (A,B) 3T3-L1 adipocytes were incubated with IL-1β (10 ng/ml, 15 min) following preincubation for 30 min in the presence or absence of A769662 (300 μmol/l). (C–E) 3T3-L1Δ1CAR adipocytes were infected with 600 ifu/cell (C) Ad.null or (D) Ad.α1DN for 48 h prior to stimulation as in (A). NFκB p65 localisation was assessed by confocal fluorescence microscopy, with Ad.α1DN-infected cells identified with anti-myc antibody. (A, C, D) Representative images are shown. (B, E) Densitometric quantification of nuclear NFκB p65 fluorescence. All data are presented as % IL-1β-stimulated nuclear fluorescence from three independent experiments with >50 cells analysed for each treatment in each experiment. Myc-positive Ad.α1DN infected cells only were analysed for filled bars in panel E. ^###^p < 0.001, ^##^p < 0.01 vs absence of IL-1β; ***p < 0.001 vs absence of A769662 (one-way ANOVA).

**Fig. 4:**
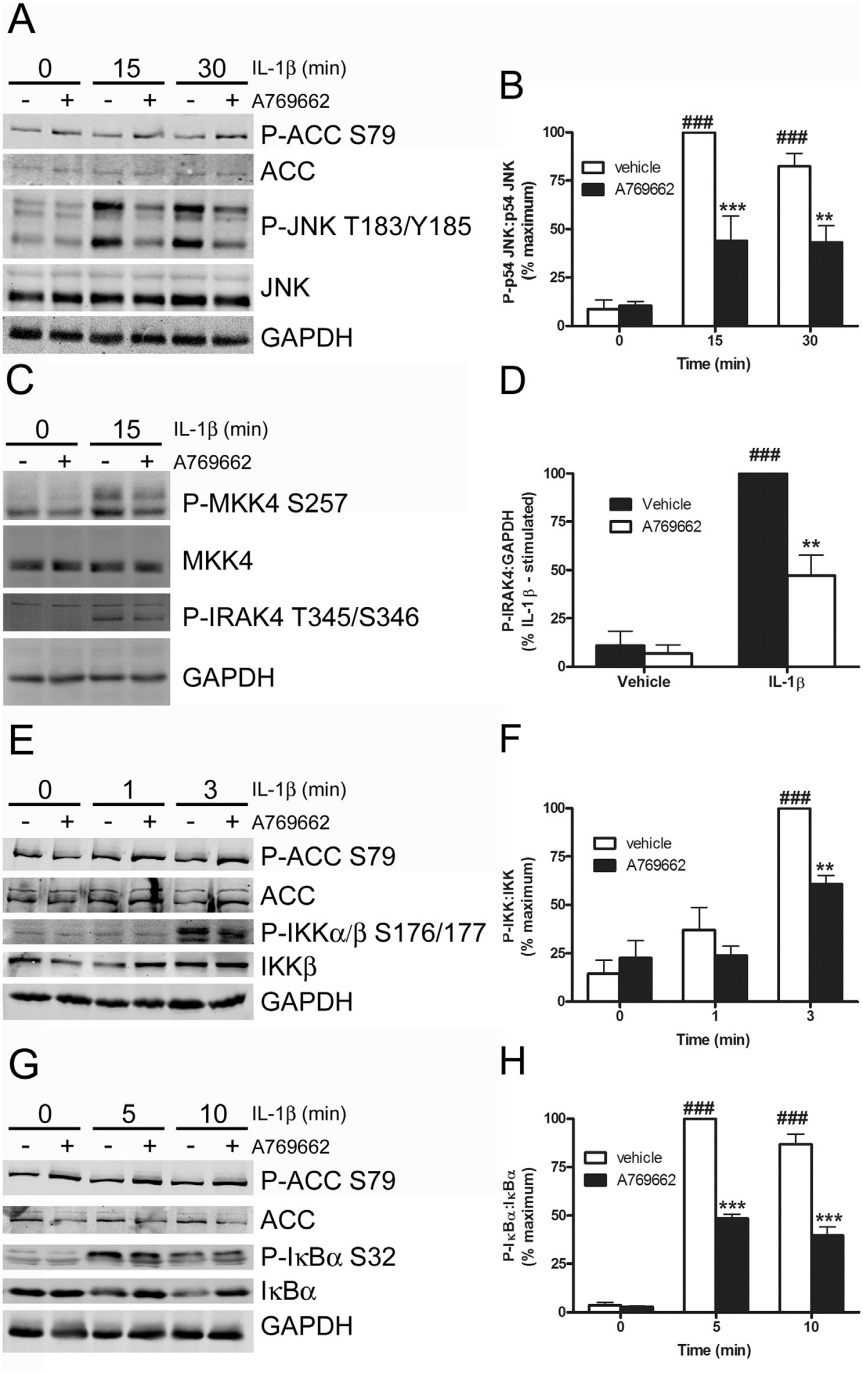
A769662 rapidly inhibits IL-1β-stimulated JNK, IRAK4, IKK and IκB phosphorylation in 3T3-L1 adipocytes. 3T3-L1 adipocytes were incubated for 30 min in the presence or absence of A769662 (300 μmol/l) prior to stimulation with IL-1β (10 ng/ml) for the indicated durations. Lysate proteins were resolved by SDS-PAGE and subjected to immunoblotting with the antibodies indicated. (A,C,E,G) Representative immunoblots. (B,D,F,H) Densitometric analysis of three independent experiments for (B) JNK (p54), D) IRAK4, (F) IKKα/β and (H) IκBα phosphorylation normalised to respective total levels in each case. ^###^p < 0.001 vs absence of IL-1β. ***p < 0.001, **p < 0.01, *p < 0.05 vs absence of A769662 (two-way ANOVA).

CXCL10, CXCL1 and MCP1 expression are stimulated by the transcription factor NFκB and/or the AP-I transcription factor complex which includes c-Jun. IL-1β stimulated the translocation of NFκB p65 to the nucleus of 3T3-L1Δ1CAR adipocytes, an effect markedly attenuated by preincubation for 30 min with A769662 (Fig. 3A and B). A769662-mediated inhibition of NFκB p65 nuclear translocation was ablated in 3T3-L1Δ1CAR adipocytes infected with a dominant-negative AMPKα1 mutant (Ad.α1-DN) (Fig. 3C–E). Efficiency of adenoviral infection with Ad.α1-DN was estimated to be approximately 60–70% and A769662-stimulated ACC phosphorylation was ablated in cells infected with Ad.α1-DN, as assessed by myc immunoreactivity (Supplemental Fig. 1A). Infection with Ad.α1-DN was not sufficient to inhibit A769662-stimulated ACC phosphorylation in total 3T3-L1 adipocyte cell lysates, however (data not shown). Furthermore, IL-1β-stimulated p65 translocation was not observed in 3T3-L1Δ1CAR adipocytes infected with a constitutively active AMPK mutant (Supplemental Fig. 1B and C). Preincubation of 3T3-L1 adipocytes with A769662 significantly attenuated IL-1β-stimulated JNK, IKK and IκB phosphorylation (Fig. 4). Furthermore, A769662 stimulation inhibited both activating phosphorylation of the JNK kinase MKK4 in addition to autophosphorylation of the most upstream kinase in IL-1β signalling, IRAK4 (Fig. 4).

**Fig. 5:**
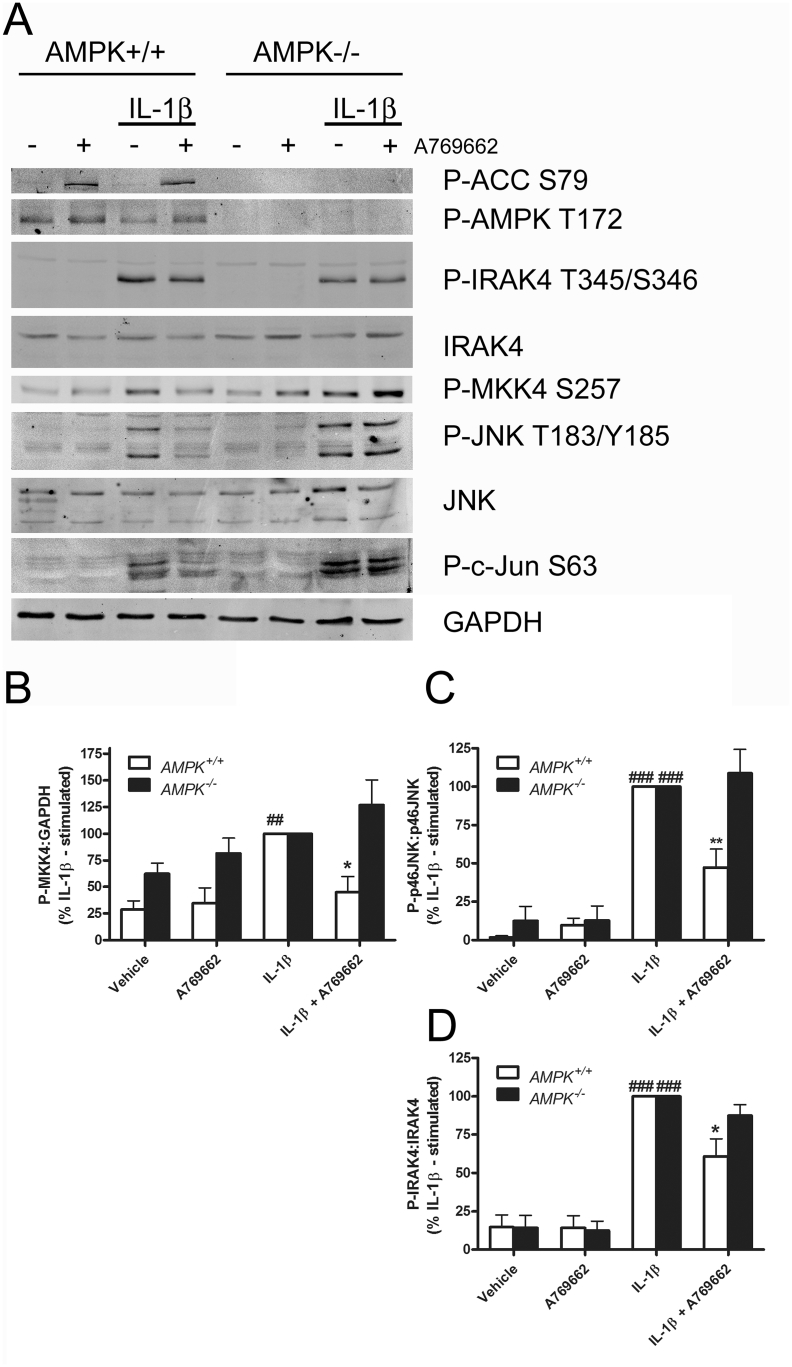
A769662-mediated inhibition of MKK4, JNK and NFκB pathway signalling is AMPK-dependent. Wild-type (AMPK+/+) and AMPK-deficient (AMPK−/−) MEFs were incubated for 30 min in the presence or absence of A769662 (100 μmol/l) prior to stimulation with IL-1β (10 ng/ml, 15 min). Lysate proteins were resolved by SDS-PAGE and subjected to immunoblotting with the antibodies indicated. A) Representative immunoblots. (B–D) Densitometric analysis of (B) MKK4 relative to GAPDH or (C) JNK (p46), (D) IRAK4 phosphorylation normalised to respective total levels in each case from three (B,D) or four (C) independent experiments. ^###^p < 0.001, ^##^p < 0.01 vs absence of IL-1β, **p < 0.01, *p < 0.05 vs absence of A769662 (one-way ANOVA).

Similar results were obtained when TNF-α was used as the pro-inflammatory stimulus at concentrations previously demonstrated to activate adipocyte proinflammatory signalling (Rotter et al., 2003), with A769662 attenuating TNF-α-stimulated CXCL10 mRNA expression (Supplemental Fig. 2), NFκB p65 nuclear translocation (Supplemental Fig. 3) and IκB phosphorylation (Supplemental Fig. 4) in cultured adipocytes. A769662 had no effect, however, on TNF-α-stimulated JNK phosphorylation (Supplemental Fig. 4). Neither IL-1β nor TNF-α had any effect on basal or A769662-stimulated AMPK activity, as assessed by ACC phosphorylation (Fig. 4, Supplemental Fig. 4). The AMPK-dependence of A769662-mediated inhibition of IL-1β-stimulated IRAK4-MKK4-JNK signalling was further investigated in AMPKα1α2^−/−^ and AMPKα1α2^+/+^ MEFs. A769662 significantly inhibited IL-1β-stimulated phosphorylation of JNK, MKK4 and IRAK4 in addition to the JNK substrate c-Jun in wild type AMPKα1α2^+/+^ MEFs, effects that were abolished in MEFs deficient in AMPKα (Fig. 5).

**Fig. 6:**
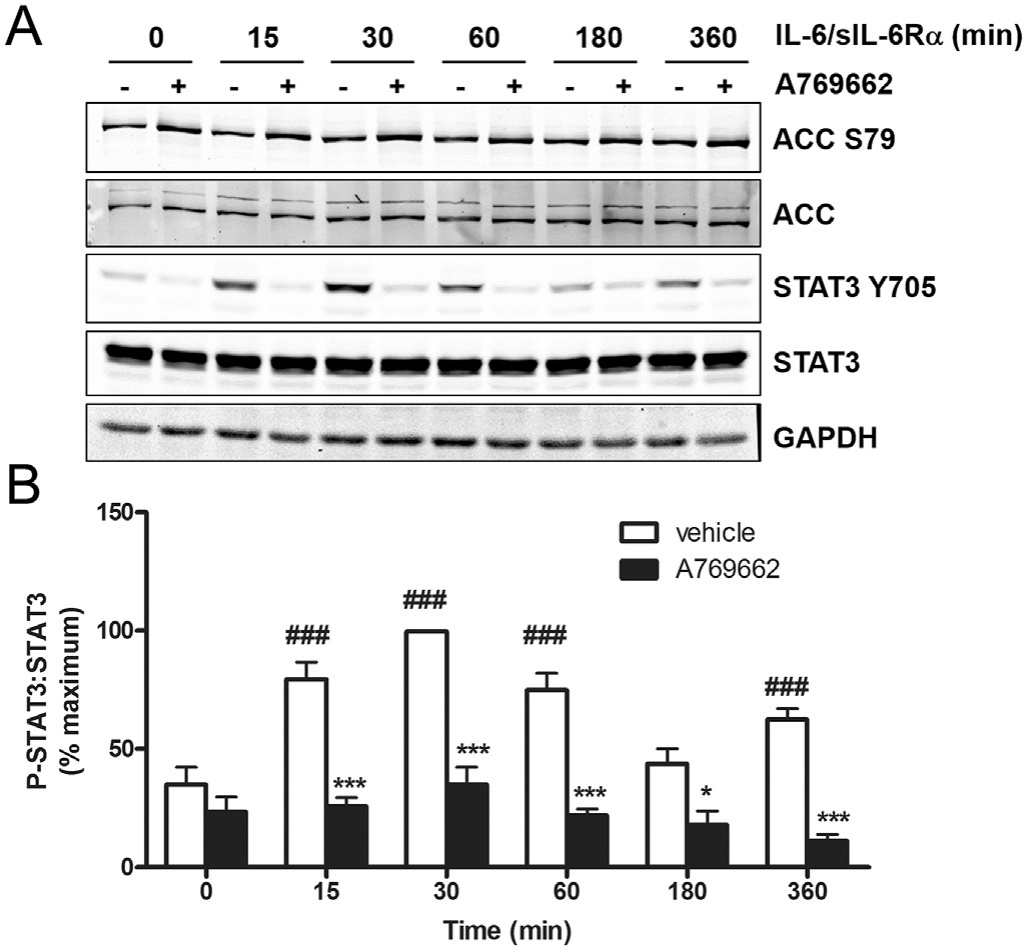
A769662 rapidly inhibits sIL-6Rα/IL-6-stimulated STAT3 phosphorylation in 3T3-L1 adipocytes. 3T3-L1 adipocytes were incubated for 30 min in the presence or absence of A769662 (300 μmol/l) prior to stimulation with IL-6/sIL-6Rα (5 ng/ml, 25 ng/ml) for the indicated durations. Lysate proteins were resolved by SDS-PAGE and subjected to immunoblotting with the antibodies indicated. (A) Representative immunoblots and (B) densitometric analysis of phospho-STAT3 relative to total STAT3 from six independent experiments. ^†††^p < 0.001, ^†^p < 0.05 vs absence of sIL-6Rα/IL-6; ***p < 0.001, *p < 0.05 vs absence of A769662 (two-way ANOVA).

Increased IL-6 concentrations have been associated with insulin resistance and obesity and, in contrast to IL-1β and TNF-α, signals *via* a JAK-STAT mediated pathway (Lee et al., 2009, Salt and Palmer, 2012). A769662 significantly inhibited STAT3 Tyr705 phosphorylation stimulated by a sIL-6Rα/IL-6 trans-signalling complex in 3T3-L1 adipocytes (Fig. 6). sIL-6Rα/IL-6 had no effect on ACC phosphorylation (Fig. 6). Stimulation of 3T3-L1 adipocytes in the presence of IL-6 but without sIL-6Rα had no effect on STAT3 Tyr705 phosphorylation (data not shown). To examine whether A769662 stimulated tyrosine phosphatases, thereby reducing IL-6-stimulated STAT3 phosphorylation, the capacity of A769662 to inhibit IL-6-stimulated STAT3 phosphorylation was examined in 3T3-L1 adipocytes preincubated with the tyrosine phosphatase inhibitor orthovanadate. Incubation with orthovanadate markedly increased IL-6-stimulated STAT3 Tyr705 phosphorylation, yet this was still markedly inhibited by A769662, indicating that AMPK-mediated inhibition is at the level of JAK activity rather than reduced STAT3 phosphatase activity (Supplemental Fig. 5).

**Fig. 7:**
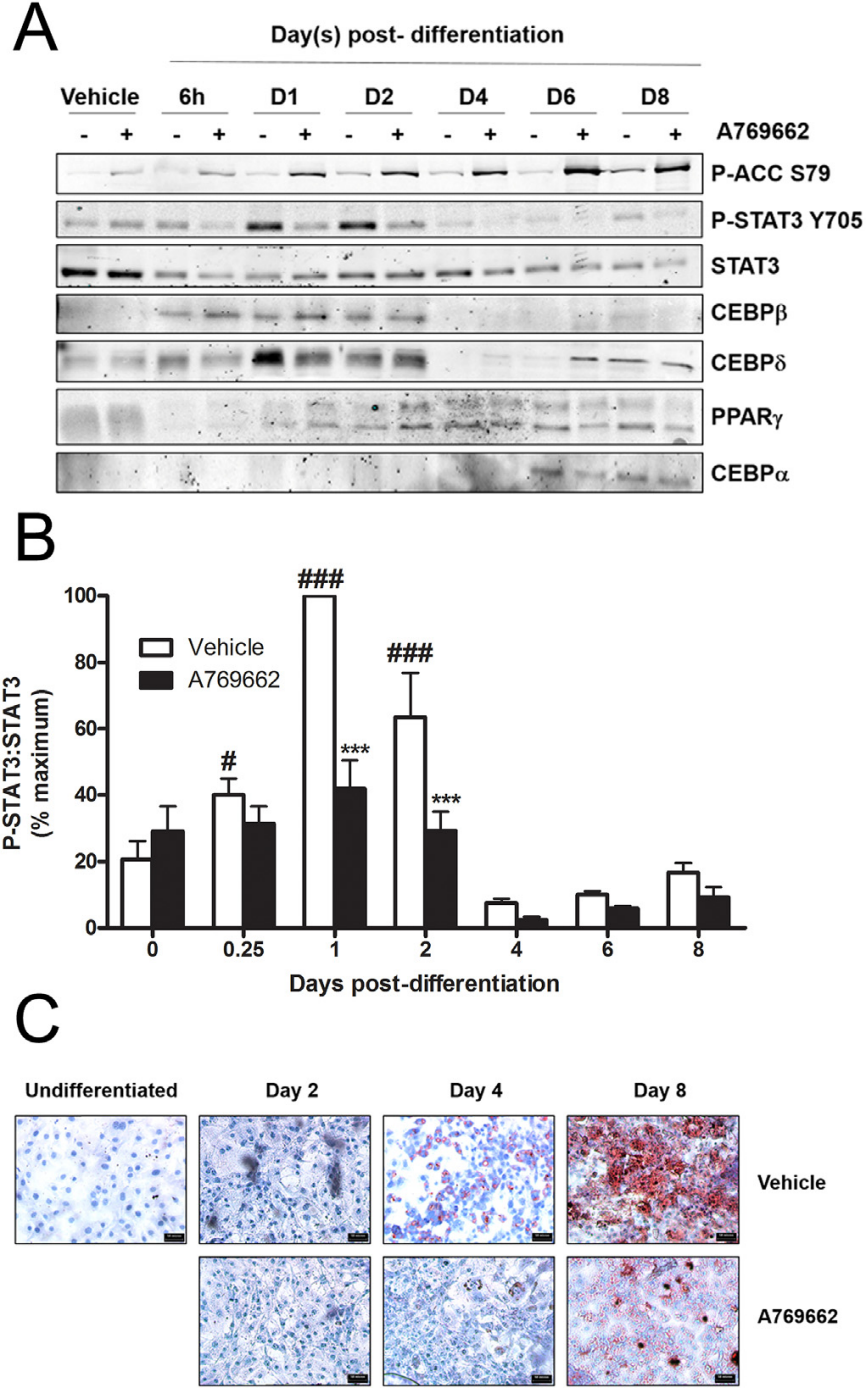
A769662 inhibits STAT3 Tyr705 phosphorylation during adipogenesis. 3T3-L1 adipocytes were differentiated in the presence or absence of A769662 (300 μmol/l). Lysates were prepared at various intervals post-differentiation and immunoblotted with the antibodies indicated. (A) Representative immunoblots. B) densitometric analysis of STAT3 phosphorylation relative to total STAT3 levels from four independent experiments. ^###^p < 0.001, ^#^p < 0.05 vs undifferentiated; *p < 0.05 vs absence of A769662 (two-way ANOVA). (C) Cells were fixed and stained with oil red O at various intervals post differentiation. Representative microscope images are shown (scale bar = 50 μm).

In contrast to IL-1β, sIL-6Rα/IL-6-stimulation of 3T3-L1Δ1CAR adipocytes did not produce detectable changes in cytokine/chemokine production (data not shown). Thus to examine the effect of AMPK activation on a JAK-STAT mediated phenomenon in adipocytes, the effect of A769662 on adipogenesis was examined. STAT3 Tyr705 phosphorylation occurred 24–48 h post-differentiation in 3T3-L1 adipocytes, an effect that was significantly reduced in the presence of A769662 (Fig. 7). A769662 had no significant effect on the early increase in expression of CEBPβ or CEBPδ during differentiation, but did suppress the later (day4 onward) increase in CEBPα and PPARγ (Fig. 7). A769662 markedly inhibited the accumulation of triglyceride during adipogenesis, as assessed by oil red O staining (Fig. 7).

**Fig. 8:**
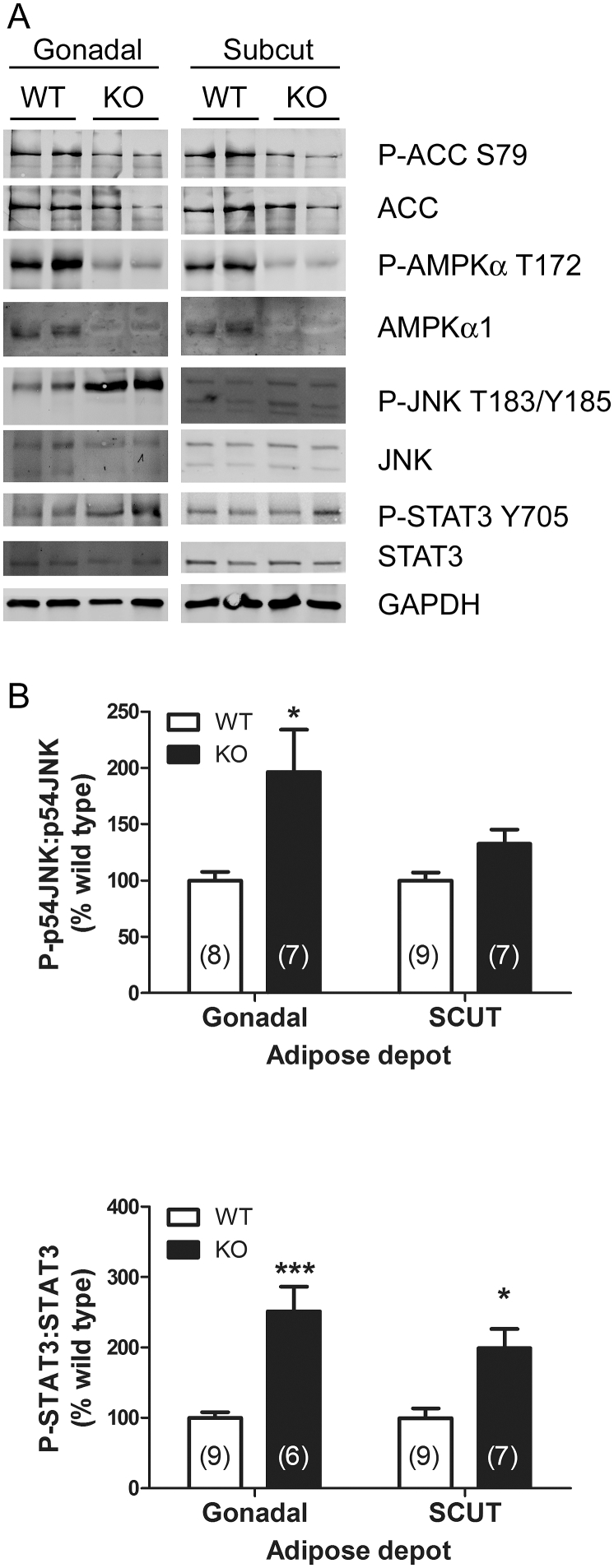
Adipose tissue from mice lacking AMPKα1 exhibit increased JNK and STAT3 phosphorylation. Gonadal or subcutaneous (Subcut) adipose tissue lysates from AMPKα1 knockout (KO) or wild-type (WT) mice were subjected to immunoblotting with the antibodies indicated. (A) Representative immunoblots shown. (B) Densitometric analysis of mean ± SEM JNK (p54) phosphorylation and STAT3 phosphorylation relative to total JNK or STAT3 levels from 8 to 9 WT and 6–7 KO animals with the number of animals in each case indicated in parentheses. ***p < 0.001, *p < 0.05 vs WT (two-tailed *t*-test).

To determine the significance of the suppressive effects of AMPK on inflammatory signalling pathway status in adipose tissue *in vivo*, JNK and STAT3 phosphorylation were assessed in subcutaneous and gonadal adipose tissue from *AMPKα1*^−/−^ mice and their wild-type littermates. Fasting blood glucose, mass and glucose tolerance were not significantly different between genotypes (Supplemental Fig. 6). Basal levels of both JNK and STAT3 phosphorylation were significantly increased in gonadal adipose tissue of AMPKα1^−/−^ mice relative to wild-type littermates. Basal STAT3 phosphorylation was also significantly increased in subcutaneous adipose tissue, with JNK phosphorylation approaching significance (p = 0.054) (Fig. 8).

## 4 Discussion

The principal findings of the study are that AMPK activation in adipocytes inhibits multiple pro-inflammatory signalling pathways, including IL-1β-stimulated phosphorylation and activation of MKK4/JNK and IKK/IκB/NFκB pathways, TNF-α-stimulated IKK/IκB/NFκB and IL-6 trans-signalling-stimulated JAK-STAT3 signalling. Furthermore, we demonstrate that AMPK-mediated suppression of IL-1β-stimulated pro-inflammatory signalling in adipocytes is associated with reduced phosphorylation of the key proximal IL-1β signalling pathway protein IRAK4 as well as markedly reduced expression and secretion of the chemokine CXCL10 by adipocytes. Increased JNK and STAT3 phosphorylation is also observed in adipose tissue depots from mice lacking AMPKα1, supporting regulation of these distinct pro-inflammatory signalling pathways by AMPK in adipose tissue *in vivo*.

### 4.1 Inhibition of IKK-IκB-NFκB signalling by AMPK

In macrophages and macrophage cell lines, AMPK-dependent inhibition of IκBα degradation and NFκB DNA binding has been reported (Sag et al., 2008, Yang et al., 2010). Furthermore, increased nuclear NFκB p65 levels have been reported in endothelial cells from mice deficient in AMPK subunits (Wang et al., 2010). Despite a clear inhibitory role for AMPK in such cell types that constitute the stromal-vascular fraction of adipose tissue, AMPK-dependent inhibition of IKK-IκB-NFκB signalling in adipocytes themselves has not been confirmed, although a compound C-sensitive inhibition of NFκB and IKKβ phosphorylation has been reported in cultured adipocytes and perivascular adipose tissue (Sun et al., 2014, Chen et al., 2016, Wang et al., 2013, Zhao et al., 2014). As compound C inhibits several protein kinases and can influence pro-inflammatory signalling independent of AMPK (Salt and Palmer, 2012, Kim et al., 2011, Bain et al., 2007), the current study compliments these findings, by providing genetic evidence of the AMPK-dependence of the inhibition of IL-1β and TNF-α-stimulated NFκB p65 translocation in adipocytes. In endothelial cells, AMPK has been proposed to inhibit NFκB signalling *via* phosphorylation of the transcriptional co-activator p300, which blocks activating acetylation of NFκB p65 (Zhang et al., 2011), however this report did not investigate the effect of AMPK activation upstream of NFκB. Alternatively, AMPK has also been proposed to hyperphosphorylate and inactivate IKK, thereby suppressing NFκB signalling (Bess et al., 2011). The findings in the current study do not fully support either of these mechanisms, as the rapid A769662-mediated inhibition of the NFκB pathway is associated with inhibition of activating IKK phosphorylation in adipocytes rather than hyperphosphorylation.

### 4.2 AMPK-mediated inhibition of JNK phosphorylation

Whether AMPK negatively regulates proinflammatory JNK signalling is far less well characterised. AMPK-dependent inhibition of JNK phosphorylation has been reported in macrophages (Jeong et al., 2009) and increased JNK phosphorylation has been reported in macrophages and endothelial cells from mice deficient in AMPK subunits (Galic et al., 2011, Dong et al., 2010). The AMPK activator AICAR has been previously reported to suppress basal JNK phosphorylation in 3T3-L1 adipocytes and adipose tissue of mice (Miyokawa-Gorin et al., 2012, Shibata et al., 2013). Despite this, the mechanism by which AMPK inhibits activating JNK phosphorylation has not been reported. In the current study, A769662 directly inhibited phosphorylation of the JNK kinase MKK4 in MEFs in an AMPK-dependent manner, demonstrating the inhibition is not due to increased JNK phosphatase activity. This is in agreement with indirect evidence in a study published when this manuscript was in preparation, in which hearts of mice with a cardiac-specific knock in of a kinase-dead AMPK were reported to exhibit increased MKK4 phosphorylation after ischaemia-reperfusion (Zaha et al., 2016). Furthermore, we show increased basal JNK phosphorylation in adipose tissue depots from mice lacking AMPKα1, reinforcing the negative regulation of pro-inflammatory JNK signalling by AMPK *in vivo*. Intriguingly, A769662 was unable to suppress TNF-α-stimulated JNK phosphorylation in 3T3-L1 adipocytes, indicating a cytokine-specific effect of AMPK activation. This is in agreement with previous studies where AICAR inhibited TNF-α-stimulated ERK1/2 phosphorylation without impairing JNK phosphorylation in 3T3-L1 adipocytes (Shibata et al., 2013) and A769662 inhibited palmitate-stimulated IKK phosphorylation without influencing JNK phosphorylation in L6 myotubes (Green et al., 2011). Taken together with the current study, this suggests inhibition of MKK4/JNK and IKK/IκB/NFκB pathways is not mediated by a common AMPK target shared by both pathways.

### 4.3 AMPK-mediated inhibition of IRAK4 phosphorylation

We demonstrate that A769662 stimulation inhibits IL-1β-stimulated phosphorylation of IRAK4 at Thr345/Ser346, a proximal event in IL-1β signalling that is not common with TNF-α signalling (Flannery and Bowie, 2010, Salt and Palmer, 2012). This shows that AMPK activation impairs IL-1β signalling at a more early stage than previously reported. It is feasible; therefore, that inhibition of JNK signalling by AMPK activation is dependent on impaired IRAK4 autophosphorylation. As IRAK4 phosphorylation is a signalling event shared by several toll-like receptors (Flannery and Bowie, 2010), AMPK activation should also inhibit signalling by other inflammatory stimuli that utilise IRAK4.

### 4.4 Inhibition of JAK-STAT signalling by AMPK

IL-6-stimulated STAT3 phosphorylation has been reported in liver-derived cells, which utilise the classical membrane-bound IL-6 receptor (Nerstedt et al., 2013, Kim et al., 2012). In contrast, IL-6 was only effective in 3T3-L1 adipocytes when administered with sIL-6Rα, demonstrating they utilise IL-6 trans-signalling. Furthermore, we demonstrate that A769662-mediated inhibition of STAT3 phosphorylation occurred 24–48 h post-differentiation in the mitotic clonal expansion (MCE) phase concomitant with reduced lipid accumulation. Transient elevation in STAT3 phosphorylation in the MCE phase is essential for adipocyte differentiation (Wang et al., 2009). This represents an earlier mechanism by which AMPK activation may inhibit adipogenesis than the reduced expression of later adipogenic markers previously reported (Habinowski and Witters, 2001). AMPK activation by A769662 rapidly attenuates IL-6-stimulated STAT3 phosphorylation in the presence of the tyrosine phosphatase inhibitor vanadate, indicating an effect on JAK activity, in agreement with studies in liver cell lines (Nerstedt et al., 2013). This is further corroborated by recent studies in our laboratories that have demonstrated that AMPK can directly phosphorylate Ser515 and Ser518 in JAK1 in vitro, and that mutation of the Ser residues to Ala ablates AMPK-mediated inhibition of JAK1-STAT signalling in intact cells (Rutherford et al., 2016). A769662-mediated inhibition of JAK activity *via* phosphorylation of Ser515/518 is therefore likely to underlie the inhibition of both IL-6 signalling and adipogenic JAK-STAT signalling.

### 4.5 Inhibition of cytokine/chemokine synthesis by AMPK activation

A769662 suppressed IL-1β-stimulated secretion of MCP-1, CXCL10 and CXCL1 from 3T3-L1Δ1CAR adipocytes in addition to IL-1β- and TNF-α-stimulated CXCL10 gene expression in SW872 adipocytes. AMPK-dependent inhibition of MCP-1 secretion has previously been demonstrated in several cell types (Salt and Palmer, 2012, Ewart et al., 2008). The current study supports and extends previous studies reporting AMPK-mediated inhibition of IFN-γ-stimulated CXCL10 mRNA expression in mouse astrocytes and microglia (Meares et al., 2013). When examining the cytokines and chemokines influenced by IL-1β in adipocytes, it has previously been reported that IL-1β stimulates MCP-1 secretion in 3T3-L1 adipocytes (Takahashi et al., 2008) and a recent study in human differentiated adipocytes reported IL-1β-stimulated secretion of MCP-1 and CXCL10 (Alomar et al., 2015). The latter study also reported IL-1β-stimulated secretion of IL-2, IL-4, IL-6, IL-10, IL-13, IL-17, IFN-γ, TNF-α, VEGF and GM-CSF, which was not observed in the current study (Alomar et al., 2015). The reason for the discrepancy may reflect the two different cell models, shorter exposure to IL-1β in our study or differential sensitivity of the assays utilised. CXCL10 is transcriptionally regulated by NFκB and pro-inflammatory MAPK signalling (Thiefes et al., 2005). As A769662 had no effect on TNF-α-stimulated JNK phosphorylation yet robustly inhibited CXCL10 mRNA expression, this may suggest that inhibition of the IKK-IκB-NFκB pathway underlies the inhibition in TNF-α-stimulated cells.

### 4.6 Increased proinflammatory signalling in adipose tissue from mice with reduced AMPK levels

As increased JNK and STAT3 phosphorylation was observed in adipose tissue depots from mice lacking AMPKα1, this indicates that these distinct pro-inflammatory signalling pathways are negatively regulated by AMPK in adipose tissue *in vivo*. In support of this, increased myocardial cytokine and chemokine expression has previously been demonstrated in the same mouse model after LPS stimulation (Castanares-Zapatero et al., 2013). As the mouse adipose used in the current study is from mice globally deficient in AMPKα1, the increased basal JNK and STAT3 phosphorylation observed may be a consequence of impaired AMPK activity in adipocytes, macrophages or other stromal-vascular cells. The effect was not due to differences in mass or glucose tolerance, which was unaltered between genotypes. Mice with adipocyte-specific deletion of AMPKα1, AMPKα2, AMPKβ1 and AMPKβ2 have recently been described (Wu et al., 2015, Mottillo et al., 2016) which would prove useful in order to further characterise the anti-inflammatory actions of AMPK in adipose.

### 4.7 Activation of AMPK in adipocytes

The concentration of A769662 (300 μmol/l) required for activation of AMPK in 3T3-L1 adipocytes and SW872 adipocytes is higher than that previously reported in several other tissues and cell types (Göransson et al., 2007, Foretz et al., 2010), although this concentration does not inappropriately alter AMP:ATP ratios in HEK293 cells (Hawley et al., 2010). As a few studies have reported AMPK-independent effects of A769662 in other cell types (García-García et al., 2010, Benziane et al., 2009, Moreno et al., 2008), the demonstration of AMPK-dependent inhibition of signalling using dominant negative mutant AMPK and MEFs lacking AMPK establishes the AMPK dependence of these effects.

Reduced AMPK activity has been reported in metabolic tissues of animal models of insulin resistance and obesity (Viollet et al., 2010, Coughlan et al., 2014). Furthermore, reduced AMPK activity has been reported in adipose tissue of insulin resistant volunteers compared with BMI-matched insulin sensitive controls (Gauthier et al., 2011) and weight loss is associated with increased adipose tissue AMPK activity and insulin sensitivity (Fritzen et al., 2015, Xu et al., 2015), although SCAT TNF-α and IL-1β mRNA levels were unchanged after weight loss (Xu et al., 2015). Multiple lines of evidence suggest activation of AMPK is a useful therapeutic aim in obesity and insulin resistance. Activation of adipose tissue AMPK is feasible, as previous work in our laboratory has shown that metformin stimulated AMPK activity in SCAT of individuals with type 2 diabetes (Boyle et al., 2011).

### 4.8 Conclusions

Taken together, our data demonstrate that AMPK activation is associated with multiple anti-inflammatory actions in adipocytes including inhibition of IL-1β-stimulated IRAK4 phosphorylation. Such anti-inflammatory actions would be likely to improve the insulin resistance associated with obesity in addition to the well-characterised metabolic actions of AMPK. Accordingly AMPK in adipose tissue is a therapeutic target worthy of further investigation.

## Funding

This work was supported by a project grant (BDA09/0003904; Alec and Beryl Warren award to IPS and DG), Ph.D Studentship (BDA09/0003948 to IPS and SJM), equipment grant (BDA11/0004309 to IPS and TMP) and Sir George Alberti Fellowship (BDA13/0004652 to ADW) from Diabetes UK and British Heart Foundation (BHF) Project Grants (PG/12/1/29276 and PG/13/82/30483 to IPS and TMP). RMT is supported by a BHF Chair (CH/12/29762).

## Duality of interest

All authors declare that there is no duality of interest associated with their contribution to this manuscript.

## Contribution statement

SJM contributed to study design, performed data acquisition and analysis, and drafted the article. ADW and SB performed data acquisition, data analysis and contributed to the writing of the manuscript. CR, DG, EAR and BV advised on the study concept, provided materials and critically revised the manuscript. RMT and TMP made substantial contributions to the study conception and critically revised the manuscript. IPS was responsible for study conception and design, performed data analysis, and drafted the article. IPS is the guarantor of this work and, as such, had full access to all the data in the study and takes responsibility for the integrity of the data and the accuracy of the data analysis. All listed authors approved the final version of the manuscript.
