# Supplementary material for: Activation of AMP-activated protein kinase rapidly suppresses multiple pro-inflammatory pathways in adipocytes including IL-1 receptor-associated kinase-4 phosphorylation
Source: Mol Cell Endocrinol. 2017 Jan 15;440:44–56. doi: 10.1016/j.mce.2016.11.010 (PMC5228585; doi:10.1016/j.mce.2016.11.010)
Supplement: Supplementary file 1 [file mmc1.docx]

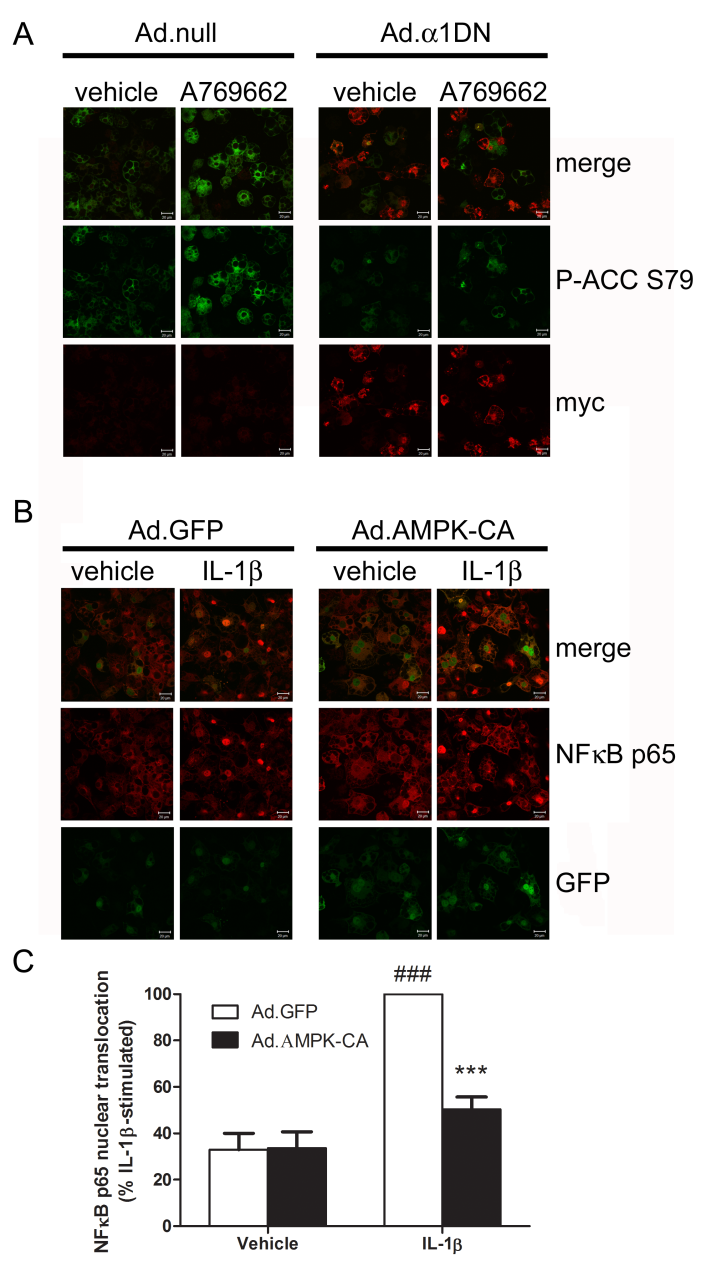


**Supplemental Fig 1. Infection with Ad.α1DN reduces A769662-stimulated ACC phosphorylation and Ad.AMPK-CA inhibits IL-1β-stimulated NFκB p65 nuclear translocation.**

3T3-L1Δ1CAR adipocytes were infected with (A) 600 ifu/cell (A) Ad.null or myc-tagged Ad.α1DN or (B) 100 ifu/cell Ad.GFP or Ad.AMPK-CA (which expresses GFP from a separate promoter) for 48 h prior to stimulation with (A) A769662 (300 µmol/l, 30 min) or (B) IL-1β (10 ng/ml, 15 min). (A) ACC phosphorylation or (B) NFκB p65 localisation was assessed by confocal fluorescence microscopy. (A,B) Representative images are shown, repeated on two further separate occasions. (C) Quantification of nuclear NFκB p65 fluorescence. All data are presented as % IL-1β-stimulated nuclear fluorescence in GFP-positive cells from three independent experiments with > 50 GFP-positive cells analysed for each treatment in each experiment. ^###^p<0.001 vs absence of IL-1β; ***p<0.001 relative to Ad.GFP (one-way ANOVA).

**
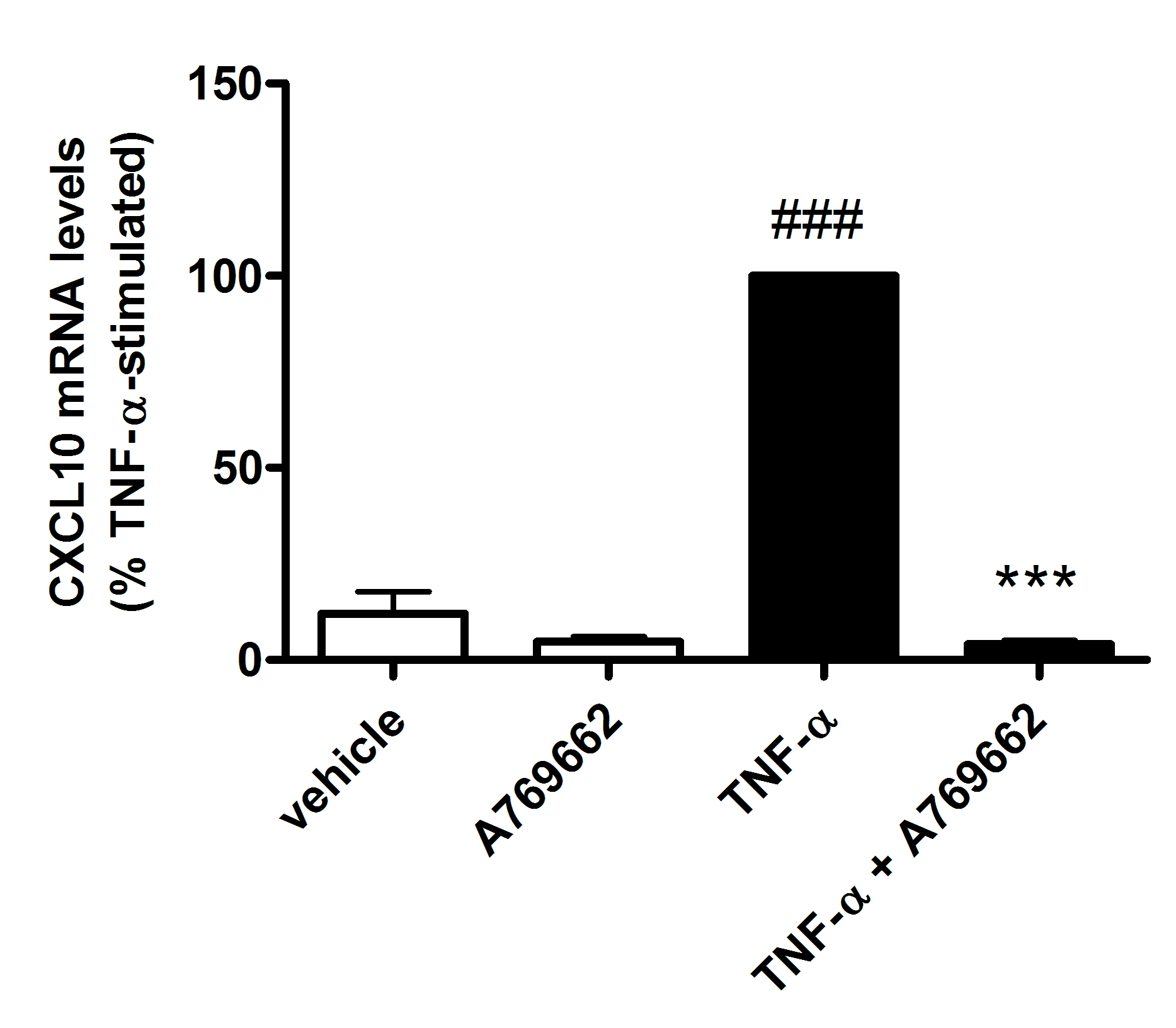
**

**Supplemental Fig 2: A769662 suppresses TNF-α-stimulated CXCL10 mRNA expression in human SW872 adipocytes**

Human SW872 adipocytes were stimulated with TNF-α (10ng/ml) for 8 h following preincubation for 30 min in the presence or absence of A769662 (300 µmol/l). CXCL10 mRNA levels were analysed by qPCR. Data shown represents the % TNF-α-stimulated CXCL10 mRNA expression normalised to TATA-binding protein from four independent experiments. ###p < 0.001 relative to absence of TNF-α. ***p <0.001 relative to absence of A769662 (one-way ANOVA).


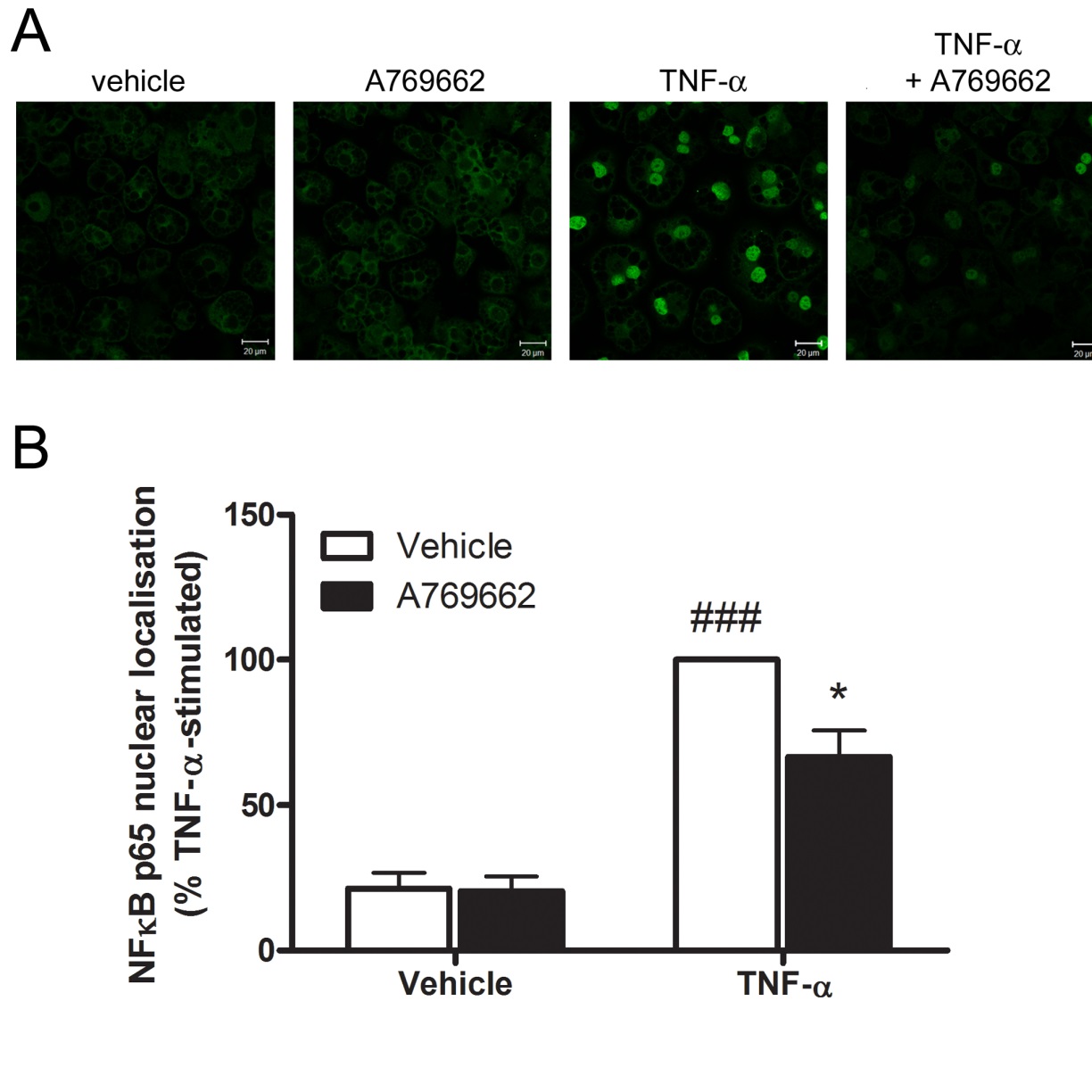


**Supplemental Fig 3. A769662 inhibits TNF-α-stimulated NFκB p65 nuclear translocation.**

3T3-L1 adipocytes were incubated with TNF-α (10 ng/ml, 15 min) following preincubation for 30 min in the presence or absence of A769662 (300 μmol/l). NFκB p65 localisation was assessed by confocal fluorescence microscopy with anti-NFκB p65 antibodies. (A) Representative images are shown. (B) Quantification of nuclear NFκB p65. All data are presented as % maximum nuclear fluorescence from three independent experiments with > 50 cells analysed for each treatment in each experiment. ^###^p<0.001 vs absence of TNF-α; *p<0.05 vs absence of A769662 (one-way ANOVA).

***
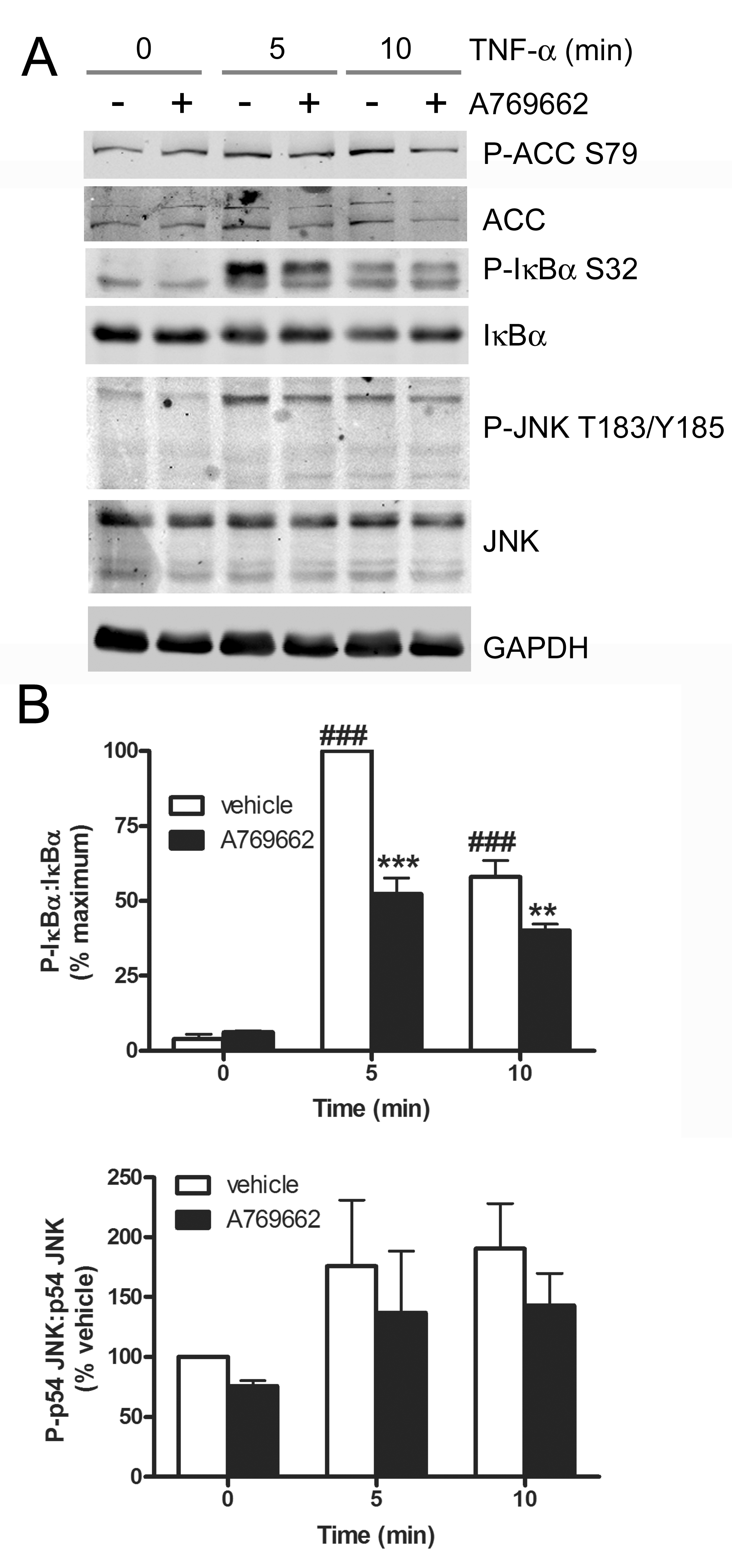
***

**Supplemental Fig 4. A769662 rapidly inhibits TNF-α-stimulated IκB phosphorylation in 3T3-L1 adipocytes**

3T3-L1 adipocytes were incubated with TNF-α (10 ng/ml) for the indicated durations following preincubation for 30 min in the presence or absence of A769662 (300 μmol/l) and lysates prepared. Lysate proteins were resolved by SDS-PAGE and subjected to immunoblotting with the antibodies indicated. (A) Representative immunoblots. (B) Densitometric analysis of IκB phosphorylation from three independent experiments. ^###^p<0.001 vs absence of TNF-α. ***p<0.001, **p<0.01 vs absence of A769662 (two-way ANOVA).

**
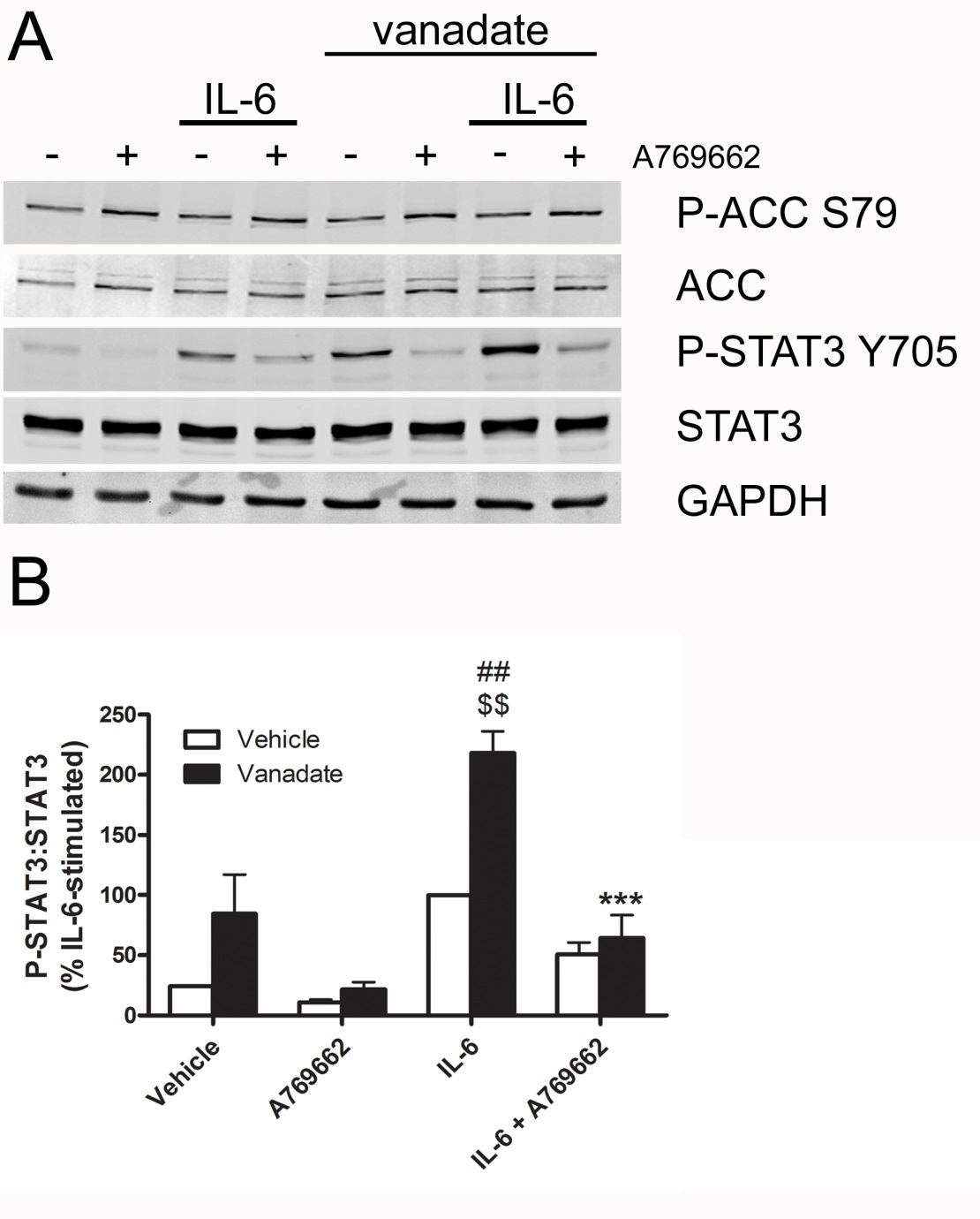
**

**Supplemental Fig 5. A769662-mediated inhibition of IL-6-stimulated STAT3 phosphorylation is unaffected by tyrosine phosphatase inhibition**

3T3-L1 adipocytes were incubated with IL-6/sIL-6Rα (5 ng/ml; 25 ng/ml) for 60 min following preincubation for 30 min in the presence or absence of A769662 (300 μM) and 30 min in the presence or absence of sodium orthovanadate (2 mM) and lysates prepared. Lysates were resolved by SDS-PAGE and subjected to immunoblotting with the antibodies indicated. (A) Representative immunoblots. (B) Densitometric analysis of STAT3 phosphorylation from three independent experiments. ***p<0.001 vs absence of A769662; ^##^p<0.01 vs absence of IL-6; ^$$^p<0.01 vs absence of vanadate (one-way ANOVA).

**
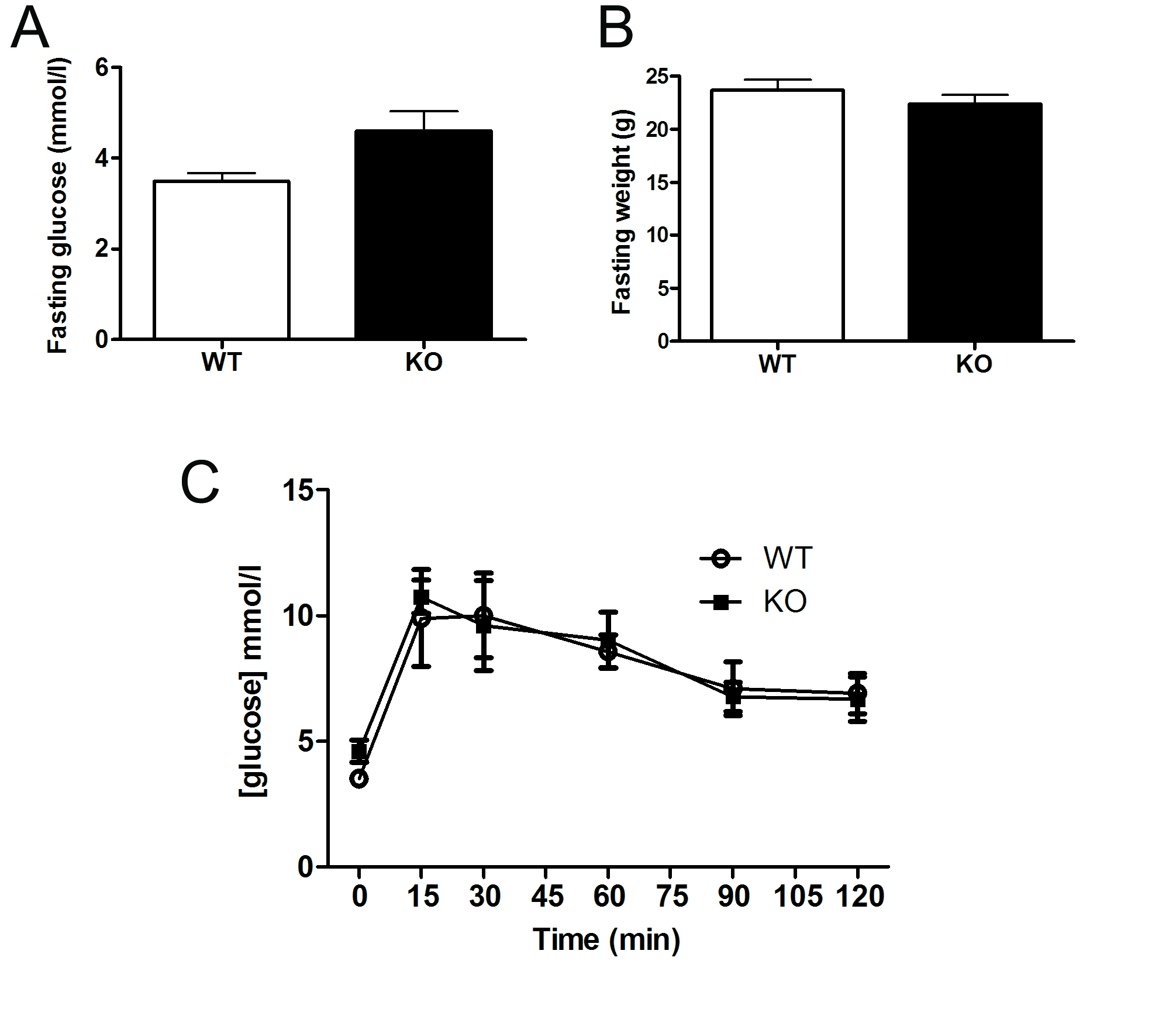
**

**Supplemental Fig 6. Basal metabolic characteristics of AMPKα1 knockout and wild type mice**

Female AMPKα1^-/-^ and wild-type littermates were weighed and glucose tolerance assessed. (A) Fasting blood glucose; (B) Mass and (C) Glucose concentrations during GTT are shown from four animals of each genotype.
